# Oral Surgery

**Published:** 1912-07-15

**Authors:** 


### BIBLIOGRAPHY.

---

ORAL SURGERY. This is a new work on oral surgery written by Stewart L. McCurdy, M. D. of the University of Pittsburgh. This is one of a series of text manuels, which it is planned shall be produced under the supervision of a committee appointed by the Institute of Dental Pedagogics. It is hoped that each subject taught in our Dental Colleges shall have a book specially written from the teacher's standpoint and directed at the dental student. The idea is concentration with comprehensive consideration of the fundamental principles involved.

Dr. McCurdy has adopted an admirable scheme of presenting first the pathologic considerations involved in inflammatory diseases with destructive tendencies or sequallae, to be followed by the more strictly operative consideration of surgical interference, rather than medical treatment. He first reviews the septic principles involved with the usual results together with a good statement of surgical disinfection and antisepsis. He then takes up the surgical treatment of dentition, alveolar abscess, stomatitis, congenital and acquired defects, tumors, fractures, dislocations, etc. in an orderly and satisfactory manner. The techique of the operations is well described and in many cases illustrated with good photographs and drawings. The printers have done their work excellently and add much to the pleasure of consulting the well printed drawings which illustrate the text. The profession is to be congratulated, and the author and committee have placed us under a great obligation. The average dentist does not feel called upon to know much of oral surgery; but we are inclined to believe that any dentist who will buy and read this excellent book will get a new interest in this phase of his work, and his patients will be better cared for. The book is published by D. Appleton & Co. and may be had of any book seller or from dental supply houses. Price $3.00.

